# Evaluation of Acidic Deep Eutectic Solvents Treatment on Enzymatic Hydrolysis Lignins: Structural Analysis and Antioxidant Activity

**DOI:** 10.3390/polym17081006

**Published:** 2025-04-08

**Authors:** Qiaorun Ma, Xun Meng, Hongtao Shi, Lian Chen, Jiangyao Han, Lifen Li, Liping Yu

**Affiliations:** College of Forestry, Guizhou University, Guiyang 550025, China; m2436563673@163.com (Q.M.); mengxun9066@outlook.com (X.M.); sht15086195477@163.com (H.S.); bigbang20060819c@163.com (L.C.); 18209828180@163.com (J.H.)

**Keywords:** acidic deep eutectic solvents, enzymatic hydrolysis lignin, antioxidant activity, ultraviolet absorption capacity

## Abstract

The high-value utilization of enzymatic hydrolysis lignin (EHL) is essential for promoting the development of the biorefinery industry. This study investigated the enhancement of the antioxidant activity and ultraviolet (UV) absorption capacity of EHL through treatment with choline chloride (ChCl)-based acidic deep eutectic solvents (DESs). The yield, chemical structure, UV absorption properties, antioxidant activity, and thermal stability of the degraded and regenerated enzymatic hydrolysis lignin (DEHL) were analyzed. The results indicated that treatment with DESs effectively preserved the aromatic structure of EHL. Compared to untreated EHL, DEHL exhibited an increased O/C atomic ratio, a decreased UV transmittance, a significant reduction in weight-average molecular weight (*M*_w_), and a notable increase in phenolic hydroxyl (ArOH) content. Notably, DEHL treated with ChCl–*p*-toluenesulfonic acid had the lowest *M*_w_ (1586 g/mol) and the highest ArOH content. Except for the ChCl–malic acid and ChCl–acetic acid systems, all the other five DES treatments enhanced the antioxidant activity of DEHL to varying degrees. Among them, DEHL treated with ChCl–*p*-toluenesulfonic acid exhibited the highest antioxidant activity, with an *IC*_50 DPPH_ value of 262.87 μg/mL.

## 1. Introduction

Lignins are one of the main components of lignocellulosic biomass and the third most abundant organic polymer on earth [1]. According to the available research [2], the annual production of lignins in nature can reach 500–3600 million tons, while the pulp and paper and biomass energy industries can produce 40–50 million tons of lignins per year [3]. However, the diversity of lignins in terms of molecular weight distribution, functional group types, and linkages, as well as the presence of organic and inorganic impurities, results in very complex structures and properties, limiting their large-scale industrial applications [4]. Currently, the effective utilization rate of lignins is less than 5% [5], and most lignins are used as low-value fuels, which is not only a huge waste of resources but also pollutes the ecological environment [6].

In order to increase the utilization value of lignin, various strategies can be employed, including reducing its molecular weight, increasing its hydroxyl content, or converting it into valuable small molecule aromatic substances [7]. Today, lignin degradation methods can be mainly divided into physical (microwave radiation and ultrasonic degradation), chemical (reduction, oxidation, hydrothermal, ionic liquid-assisted, etc.), and biological degradation (fungi and bacteria) [8,9]. At present, physical methods often face challenges such as high energy consumption, low conversion efficiency, and a wide range of reaction products. Biological methods mainly suffer from the disadvantages of long processing times and limited degradation efficiency. Among these approaches, chemical degradation is one of the most widely used techniques in lignin decomposition. In recent years, ionic liquids (ILs) have attracted much attention as solvents for the catalytic degradation of lignin. These solvents are considered “green” due to their non-volatility, non-flammability, tunable nature, and high solubility for lignin [10]. The catalytic or oxidative depolymerization of lignin model compounds and lignin itself using ILs can yield valuable platform aromatic compounds such as vanillin, vanillic acid, and other potential substitutes for petroleum-based feedstocks [11]. However, the industrial application of certain ILs is limited by their high cost, complexity of synthesis, high viscosity, and, in some cases, toxicity and non-biodegradability [12].

Deep eutectic solvents (DESs) have a structure similar to that of ILs and are liquid eutectic mixtures composed of hydrogen bond acceptors and hydrogen bond donors. Typically, they consist of two or three components that can bond with each other through hydrogen bonds. The concept of DESs was first introduced by Abbott et al. [13], who prepared DESs using quaternary ammonium salts and amide compounds with melting points lower than any of the individual components. Due to their simple synthesis, renewability, mild reaction conditions, low toxicity, good solubility for lignin [14], and better preservation of lignin structure during the separation and extraction of lignin from lignocellulosic biomass [15,16], DESs have been favored in recent years in the research and application of lignin extraction, modification, and degradation. They are considered ideal alternatives for lignin extraction and degradation, and they are also regarded as promising substitutes for ILs [17].

Three types of DESs, namely acidic, neutral, and basic, can be formed by using choline chloride (ChCl) as a hydrogen bond acceptor and various compounds as hydrogen bond donors. In acidic DES, the hydrogen bond donors mainly consist of monoacid and some polyacids compounds. While in basic DESs, the hydrogen bond donors primarily include urea, amine, and amides. Neutral DESs usually use alcohols as hydrogen bond donors. Acidic DESs have been reported to outperform other types of DESs in terms of lignin extraction efficiency [18]. In recent years, acidic DESs have gained significant attention for extracting lignin with excellent antioxidant properties from lignocellulosic materials such as wood [19], bamboo [20], crop stalks [21], and fruit shells [22]. The efficiency and antioxidant activity of the extracted lignin are influenced by various factors, including the types of DESs, extraction temperature, extraction time, and solid-to-liquid ratio. Generally, the antioxidant activity of extracted lignin increases with higher temperatures and longer extraction times. Among these, ChCl–lactic acid is considered a promising DES for the pretreatment of lignocellulosic materials [23]. Moreover, there has been growing interest in utilizing acidic DESs for industrial lignin degradation under mild conditions. For example, Li et al. [24] used ChCl–*p*-toluenesulfonic acid to degrade alkaline lignin and found that this DES selectively cleaved most of the ether bonds in lignin. This led to a decrease in molecular weight and an increase in phenolic hydroxyl (ArOH) content of the regenerated lignin. Similarly, Hong et al. [25] used ChCl–formic acid to degrade alkaline lignin at 80–120 °C, demonstrating that a large number of *β*-*O*-4 linkages were broken, along with some C-C bonds (i.e., *β*-5′, *β*-*β*′). They also observed the dehydration of hydroxyl groups on lignin side chains and a partial loss of methoxy groups. In another study, Li et al. [26] compared the degradation performances of seven different ChCl-based DESs on alkaline lignin and found that the regenerated lignin had a lower molecular weight and was more uniformly dispersed compared to untreated alkaline lignin. This improvement was mainly attributed to the effectively cleaving the *β*-*O*-4 aryl ether bond in lignin by the acidic DES treatment.

Enzyme hydrolyzed lignin (EHL) can be produced by using enzymes to degrade cellulose and hemicellulose in agricultural and forestry waste, as well as food residues, during the production of bioenergy or chemicals [27]. After enzymatic treatment, the fermentation processes yield valuable products such as ethanol, butanol, and functional sugars. The remaining residues are then separated and purified to produce EHL. It has been reported that EHL can be produced in quantities of up to 200,000 tons per year [28], and this production is expected to increase significantly each year with the rapid development of biochemicals and bioenergy research [29]. Since EHL is produced through bioextraction methods, it avoids the high-temperature and high-pressure cooking processes typically used in the production of alkali lignin and sulfate lignin. As a result, EHL retains structurally active functional groups, such as ArOH and aliphatic hydroxyl (AlkOH) groups, which remain more intact compared to other types of lignin. However, current challenges in utilizing EHL include its high molecular weight and low antioxidant activity. Presently, a large amount of EHL is burned as fuel, and there remains substantial potential for its resource utilization [30].

Currently, DESs are primarily used for the pretreatment of lignocellulosic materials, with relatively limited research on the degradation of industrial lignin, such as EHL. In this study, seven acidic DESs were prepared using ChCl as the hydrogen bonding acceptor and various acids as hydrogen bonding donors. These acids include monoacids (lactic acid, acetic acid, and formic acid), dibasic acids (oxalic acid and malic acid), a ternary acid (citric acid), and a Brønsted acid (*p*-toluenesulfonic acid). EHL was treated with these seven DESs, and the yields of DEHL were measured using a gravimetric method. To investigate the effects of different acidic DESs treatments on the chemical structure, molecular weight, and thermal stability of lignins, a range of analytical techniques, including Fourier transform infrared (FTIR) spectroscopy, X-ray photoelectron spectroscopy (XPS), ultraviolet (UV–Vis) spectroscopy, gel permeation chromatography (GPC), and thermogravimetric analysis (TG), were employed. Additionally, the DPPH radical scavenging activity of EHL and DEHL was tested, and the relationship between the ArOH content of lignins and their antioxidant activity was further explored. This work aims to address the challenges associated with the low degradation and utilization efficiency of industrial lignins by developing DEHL with improved antioxidant properties. This approach not only provides new pathways for the high-value utilization of biomass resources but also promotes the sustainable development of the biorefinery industry.

## 2. Materials and Methods

### 2.1. Materials

Enzymatic hydrolysis lignins (EHLs) were purchased from Shandong Longli Bio-technology Co., Ltd., Dezhou, China, and was extracted from corn kernel residue after functional sugar preparation. Lactic acid (analytically pure, 85.0–90.0%) was purchased from Sailanbo Technology Co., Ltd., Guizhou, China; ChCl (analytically pure, 98%), acetic acid (analytically pure, ACS, ≥99.7%)), formic acid (ACS, ≥96%), oxalic acid (analytically pure, 98%), malic acid (analytically pure, >99.0%), citric acid (analytically pure, 99.5%), *p*-toluenesulfonic acid monohydrate (analytically pure, ≥98.5%), sodium hydroxide (97%, in flakes), methanol (chromatographic grade, ≥99.9%), 1,4-dioxane (ACS, ≥99.0%), diphenylpropylphenylhydrazine (DPPH) (97%), acetyl chloride (AR, 98%), glacial acetic acid (AR, 99.5%), gallic acid (99%), and anhydrous sodium carbonate (≥99.8%) were purchased from Aladdin Biochemistry and Technology Co., Ltd., Shanghai, China. All materials were used directly without treatment.

### 2.2. Preparation of Series DESs

All raw materials for the preparation of DESs were dried in a vacuum oven at 80 °C for 12 h. The molar ratio of ChCl to *p*-toluenesulfonic acid was 1:1, while the molar ratio of ChCl to the other acids (lactic, acetic, formic, oxalic, malic, and citric) was 1:2, and the mixtures were stirred at 100 °C until a clear liquid formed; these were labeled ChCl-LA, ChCl-HAc, ChCl-FA, ChCl-OA, ChCl-MA, ChCl-CA, and ChCl-TsOH. The prepared DESs were sealed and stored in a desiccator. The pH values of the different DESs were measured using a pH meter (PHS-3C, Shanghai Yifen Scientific Instrument Co., Ltd., Shanghai, China) at 20 °C. Each group tested three times, and the average value reported.

### 2.3. Purification of EHLs

The precisely weighed EHLs (15.0 g) were dissolved in 300 mL of a sodium hydroxide aqueous solution with a concentration of 1 mol/L. The mixture was then filtered to remove insoluble and poorly soluble impurities. Subsequently, 1 mol/L sulfuric acid solution was gradually added to the lignin filtrate, adjusting the solution pH to a range of 2 to 3, which caused the lignin to precipitate. The precipitate was filtered and washed with a large amount of distilled water to remove salts and other impurities, ensuring that the final filtrate had a pH of 6 to 7. The filter residue was collected and dried in an oven at 80 °C for 12 h. After drying, the residue was ground and passed through a 100-mesh sieve. The resulting sample was the purified EHLs.

### 2.4. Degradation of EHLs with DESs

A total of 20.0 g (accurate to 0.1 g) of DESs was placed in a reagent bottle and heated at 120 °C using a thermostatic magnetic stirrer (DF-101S, Shanghai Qiuzuo Scientific Instrument Co., Ltd., Shanghai, China). Approximately 1.0 g (*m*_0_) of the purified EHLs was then added, and the mixture was stirred for 5 h. At the end of the mixing process, the DESs–EHLs mixture was slowly dropped into 100 mL of rapidly stirred distilled water, and stirring continued for an additional 30 min. After the designated time, the filtrate was separated from the residue using a 0.45 μm filter membrane, and the filtrate was washed three times with 60 mL of distilled water. Finally, the filtrate was dried to absolute dryness at 80 °C to obtain degraded and regenerated EHLs (DEHLs), and the mass of DEHLs was weighed (*m*_1_). The DEHLs obtained using ChCl-LA, ChCl-HAc, ChCl-FA, ChCl-OA, ChCl-MA, ChCl-CA, and ChCl-TsOH are denoted as ChCl-LA-DEHL, ChCl-HAc-DEHL, ChCl-FA-DEHL, ChCl-OA-DEHL, ChCl-MA-DEHL, ChCl-CA-DEHL, and ChCl-TsOH-DEHL. The DEHL yields after treatment with different acidic DESs were calculated using the gravimetric method according to Equation (1).

(1) $$DEHL\;yield=\frac{m_{1}}{m_{0}}\times 100\%$$

### 2.5. Analysis of the Structure and Antioxidant Activity of EHLs and DEHLs

#### 2.5.1. Chemical Structure

The chemical structures of EHLs and DEHLs were analyzed by the KBr pellet method using a Fourier transform infrared spectrometer (Nicolet iS20, Thermo Fisher Scientific, Waltham, MA, USA). The test was performed with a scanning range of 400 to 4000 cm^−1^, a resolution of 2 cm^−1^ and 32 scans.

#### 2.5.2. Surface Elements Analysis

The surface elements of EHLs and DEHLs were analyzed using an X-ray photoelectron spectrometer (K-Alpha, Thermo Fisher Scientific, Waltham, MA, USA). The fluence energies and step sizes for full-spectrum and narrow-spectrum scanning were set at 150 eV and 1 eV and 50 eV and 0.1 eV, respectively.

#### 2.5.3. UV Absorption and Shielding Capacity

The UV absorption and transmission spectra of EHLs and DEHLs were analyzed using a UV–visible spectrophotometer (UV-9000, Shanghai Yuananalytical Instrument Co., Ltd., Shanghai, China) according to the method described in the literature [31], with slight modifications.

For UV absorption spectroscopy, 3.75 mg of lignins was dissolved in a 10 mL mixture of dioxane/water (9:1, *m*/*m*) with stirring to ensure complete dissolution. Subsequently, 1 mL of the lignin solution was diluted with 9 mL of the same dioxane/water mixture, resulting in a final lignin concentration of 0.0375 mg/mL for testing. The absorption spectra of both EHLs and DEHLs were then recorded over a wavelength range of 230 to 600 nm.

For UV transmission spectroscopy, 4.00 mg of lignin was dissolved in a 10 mL mixture of dioxane/water (9:1, *m*/*m*) with stirring. The appropriate amount of the lignin solution was then diluted to achieve a concentration of 0.20 mg/mL, and the transmittance was measured over a wavelength range of 230 to 600 nm.

#### 2.5.4. Molecular Weight Distribution

Prior to the determination of molecular weight of the lignins, they were modified through acetylation according to previous studies [32,33] to improve their solubility in tetrahydrofuran. The method was as follows: Approximately 0.2 g of lignins was weighed and placed in a round-bottom flask. Next, 20 mL of acetylation reagent was added, which consisted of a mixture of acetyl chloride and glacial acetic acid in the ratio of 1:4 (*v*/*v*). The mixture was then stirred in a sealed environment at 40 °C for 2 h. Upon the completion of the reaction, the untreated solvent was removed using a rotary evaporator (RE-52AA, Shanghai Cancun Instrument Co., Ltd., Shanghai, China). Subsequently, 20 mL of distilled water was added to the solid residue and ultrasonicated for 5 min. The resulting mixture was vacuum-filtered through a 0.45 μm membrane. The filter residue was washed thoroughly with a large volume of distilled water until no acidic odor remained. Finally, the product was vacuum-dried to obtain acetylated lignin.

The molecular weights of EHLs and DEHLs were determined using gel permeation chromatography (Waters1525/Waters2414, Waters Technology (Shanghai) Co., Ltd., Shanghai, China). Specifically, 0.02 g of dried acetylated lignins was dissolved in tetrahydrofuran to make a solution with a concentration of 2 mg/mL. Prior to analysis, the solution was filtered through a 0.22 μm filter membrane. GPC analysis was performed using an Agilent PL gel 5 µm MIXED-C, with tetrahydrofuran as the mobile phase at a flow rate of 1 mL/min, an injection volume of 20 µL, and the column temperature maintained at 35 °C.

#### 2.5.5. ArOH Content Test

The ArOH content of EHL and DEHL was analyzed using the Folin–Ciocalteu method, with gallic acid as the standard [34]. A series of gallic acid solutions were prepared by dissolving various amounts of gallic acid in a 90% (*v*/*v*) 1,4-dioxane/water solution at concentrations of 40, 80, 120, 160, 200, and 240 μg/mL. At each concentration, 0.2 mL of the gallic acid solution was mixed with 0.5 mL of Folin–Ciocalteu reagent and was shaken thoroughly. After 5 min of reaction, 1 mL of 15% (*m*/*m*) Na_2_CO_3_ solution was added, and the volume was adjusted to 10 mL with water. The mixture was then shaken well and incubated at 25 °C for 2 h protected from light. The absorbance at 760 nm was measured using a UV–visible spectrophotometer (UV-2700, Shimadzu (Hong Kong) Ltd., Hong Kong, China), and a standard curve was plotted. It was found that there was a strong linear relationship between the concentration of gallic acid (from 40 to 240 μg/mL) and the absorbance, which can be expressed by the regression equation y = 0.00275x − 0.00514, with an R^2^ value of 0.9994, indicating a highly significant correlation. This standard curve allows for the calculation of total ArOH content in EHL and DEHL.

A 0.2 mL aliquot of lignin solution in 90% (*v*/*v*) 1,4-dioxane/water at a concentration of 0.5 mg/mL was thoroughly mixed with 0.5 mL of flufenoxuron reagent. The reaction was carried out for 5 min, after which 1 mL of 15% Na_2_CO_3_ solution was added. The total volume was then adjusted to 10 mL with distilled water, and the mixture was shaken well and incubated at 25 °C for 2 h protected from light. The absorbance of the solution at 760 nm was measured using a UV–vis spectrophotometer. The ArOH content of the lignins was determined based on the gallic acid standard calibration curve. The total ArOH content of lignin samples was expressed as gallic acid equivalent (GAE), i.e., mg GAE/100 mg lignin. Three measurements were taken for each specimen, and the average value was used for the analysis.

#### 2.5.6. Thermal Stability

The thermal stability of the lignins was determined using a thermogravimetric analyzer (Netzsch STA 449F3, Selb, Germany) under a N_2_ atmosphere. The temperature range analyzed was from 30 to 800 °C, with a heating rate of 10 °C/min and an N_2_ flow rate of 60 mL/min.

#### 2.5.7. Antioxidant Properties

The antioxidant activity of the lignins was evaluated using the DPPH radical scavenging method based on the literature [35]. The method is as follows: An appropriate amount of purified EHL and DEHL was dissolved in 0.1 mL of a 90% aqueous dioxane solution at lignin concentrations of 100, 200, 300, 400, 600, and 800 μg/mL, respectively. Subsequently, 3.9 mL of DPPH solution (0.06 mmol/L in methanol) was added to the mixture. The reaction was carried out at 25 °C for 30 min protected from light. At the end of the reaction, the absorbance (*A*_s_) of the mixture at 517 nm was measured by a UV–Vis spectrophotometer (UV-2700, Shimadzu (Hong Kong) Ltd., Hong Kong, China). Simultaneously, the absorbance (*A_b_*) of the control mixture containing 0.1 mL of 90% dioxane and 3.9 mL of DPPH solution was measured. The scavenging rate of DPPH radicals by different lignins was calculated using Equation (2), and the half-maximal inhibitory concentration (*IC*_50_) of DPPH radicals was determined. Three measurements were taken for each sample, and the results were averaged. Lower *IC*_50_ values indicate the better antioxidant activity of the materials.

(2) $$DPPH\;\mathrm{Clearance}\;\mathrm{Rate}=\frac{A_{b}-A_{s}}{A_{b}}\times 100\%$$

## 3. Results and Discussion

### 3.1. Degraded and Regenerated EHL Yield in Different Acidic DESs

The pH values of DESs and the yields of DEHL are shown in Table 1. It was found that the seven DESs prepared with ChCl as the hydrogen bonding acceptor and lactic acid, acetic acid, formic acid, oxalic acid, malic acid, citric acid, and *p*-toluenesulfonic acid as the hydrogen bonding donors were all acidic in nature. Of these, ChCl-LA was the least acidic, with a pH value of 3.37, while ChCl-OA was the most acidic, with a pH value lower than 2.00. The yields of DEHL ranged from 92.03% to 94.14% after treatment at 120 °C for 5 h. Among these, ChCl-MA treatment showed the highest DEHL yield, followed by ChCl-TsOH, and ChCl-FA treatment showed the lowest DEHL yield. In general, the acidity of DESs seems to have little effect on the DEHL yield.

### 3.2. Infrared Spectra of Lignin

**Figure 1:**
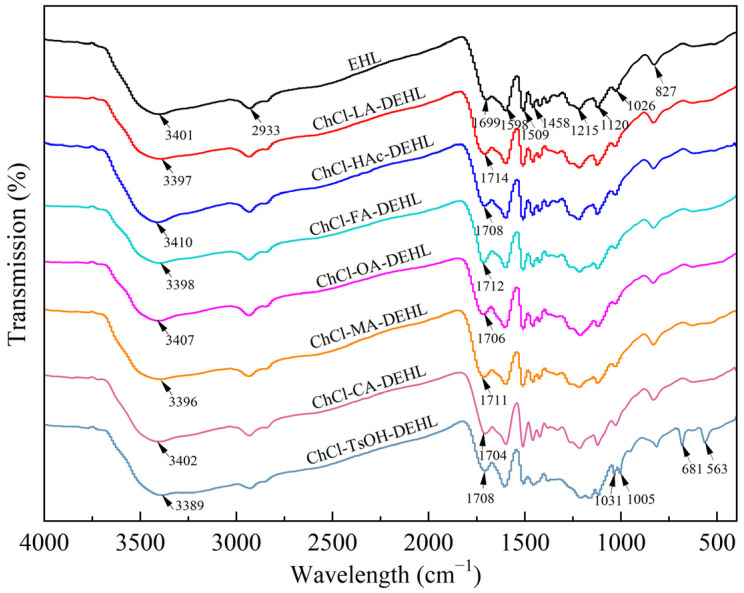
FT-IR spectra of EHL and DEHL.

The functional groups of EHL and DEHL were analyzed using FTIR spectroscopy, and the results are shown in Figure 1. It was observed that all the lignin samples showed obvious stretching vibrations of ArOH and AlkOH groups around 3401 cm^−1^, indicating the presence of a large number of hydroxyl groups in lignin. The absorption peaks at 2933 cm^−1^ and 2850 cm^−1^ are attributed to the C-H vibrations in the methoxyl and methylene groups [36]. Moreover, the absorption peak at 1699 cm^−1^ corresponds to the carbonyl group (C=O) in conjugated ketones and carboxylic acids. Additionally, the absorption peaks at 1598 cm^−1^ and 1509 cm^−1^ are associated with aryl ring vibrations, carbonyl vibrations, and stretching vibrations of the aryl ring skeleton. The absorption peaks observed at 1458 cm^−1^ and 1421 cm^−1^ are attributed to the deformation of the C-H single bonds and the bending vibrations of methoxy and methylene groups in the asymmetric -CH_3_ and -CH_2_ groups, respectively. In addition, the absorption peak at 1215 cm^−1^ corresponds to the formation of C-C bonds in the guaiacyl group, as well as the stretching vibrations of C-C, C-O, and C=O within the guaiacyl structure. The peak at 1120 cm^−1^ is associated with the in-plane deformation vibrations of C-H in guaiacyl, while the peak at 1026 cm^−1^ results from a combination of the in-plane deformation of the aromatic C-H, the deformation of C-O, and the vibration of C=O. Finally, the absorption peak at 827 cm^−1^ corresponds to the bending vibration of the aromatic cyclic C-H group. Overall, the FTIR spectroscopy analysis indicated that the absorption peaks of lignin did not change significantly before and after treatment with DESs, suggesting that DEHL basically retained the original aromatic compound structure of EHL.

In addition, the analysis showed that the hydroxyl absorption peaks of DEHL treated with ChCl-LA, ChCl-FA, and ChCl-MA exhibited a certain degree of blue shift. Specifically, the wavelengths of these hydroxyl absorption peaks shifted from 3401 cm^−1^ to 3397, 3398, and 3396 cm^−1^, respectively. Notably, the hydroxyl absorption peaks of ChCl-TsOH-DEHL underwent a significant blue shift to 3389 cm^−1^. In contrast, the hydroxyl absorption peak of ChCl-HAc-DEHL was markedly red-shifted to 3410 cm^−1^. The hydroxyl absorption peaks of ChCl-OA-DEHL and ChCl-CA-DEHL shifted to 3407 and 3402 cm^−1^, respectively, both showing similar red shifts.

Furthermore, the carbonyl (C=O) absorption peaks of all DEHLs exhibited red shifts. The carbonyl absorption peaks of ChCl-LA-DEHL, ChCl-HAc-DEHL, ChCl-FA-DEHL, ChCl-OA-DEHL, ChCl-MA-DEHL, ChCl-CA-DEHL, and ChCl-TsOH-DEHL moved from 1699 cm^−1^ to 1714, 1708, 1712, 1706, 1711, 1704, and 1708 cm^−1^, respectively, with an increase in the intensity of these absorption peaks. The changes in the wavelengths and intensities of the lignin hydroxyl and carbonyl groups peaks can be attributed to various chemical reactions that occurred in lignin during different acidic DESs treatments. These reactions include the cleavage of the *β*-*O*-4 ether bonds, the formation of Hibbertone, and the reaction of some hydroxyl groups in lignin with carboxylic acids in the DESs, resulting in the formation of the corresponding esters [37,38]. Additionally, significant absorption peaks were observed at 681 cm^−1^ and 563 cm^−1^ in ChCl-TsOH-DEHL, likely corresponding to the stretching vibrations of the *p*-toluenesulfonic acid-related group (SO_3_) [39]. This phenomenon may be due to the sulfonation reaction that occurred during the treatment of EHL with ChCl-TsOH [40].

### 3.3. X-Ray Photoelectron Spectroscopy of Lignin

**Figure 2:**
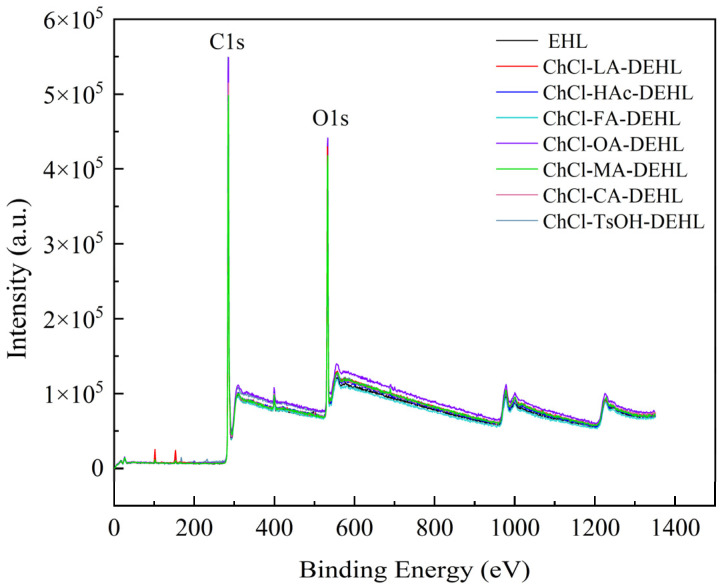
XPS wide scan of EHLs and DEHLs.

To investigate the effect of different acidic DESs treatments on the surface elements of EHLs, X-ray photoelectron spectroscopy (XPS) was used to analyze the surface elements composition of EHLs and DEHLs. The broad-scan XPS spectra of EHLs and DEHLs are shown in Figure 2. As can be seen, the XPS broad-scan spectra of all lignins showed prominent peaks around 285 eV and 532 eV, indicating that lignins are rich in carbon and oxygen. In addition, a weak absorption peak appeared near 400 eV, indicating that lignins also contain a small amount of nitrogen.

The results of the O/C atomic ratios of EHLs and DEHLs are shown in Table 2. It can be found that the O/C atomic ratio of EHLs was 0.239, which closely aligns with the findings of Zhou et al. [41]. Compared to EHLs, the O/C ratios of DEHLs increased, with ChCl-TsOH-DEHLs showing the smallest increase, resulting in an O/C ratio of 0.250. Conversely, ChCl-LA DEHLs showed the largest increase, with an O/C ratio of 0.283. The treatment of DESs, such as ChCl-MA, also markedly increased the O/C atomic ratios of DEHLs. This increase can be attributed to the esterification reactions that occurred during the treatment of EHLs with carboxylic acid-based DESs, such as ChCl-LA and ChCl-MA [42]. Similarly, Chirila et al. [43] also found that the relative oxygen content on the surface of modified lignins increased, while the relative content of carbon decreased when lignins were modified with carboxylic acids, including oleic, lactic, and butyric acids.

### 3.4. UV–Vis Absorption and Transmission Spectra of Lignins

**Figure 3:**
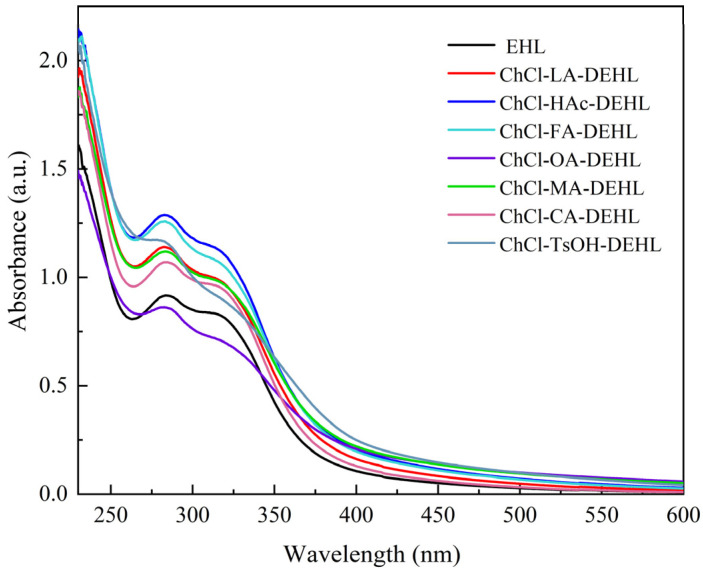
Ultraviolet–visible absorption spectra of EHLs and DEHLs.

As an aromatic compound, lignins exhibit UV absorption capacity. The UV–visible absorption spectra of EHLs and DEHLs were analyzed using a UV–visible spectrophotometer, and the results are shown in Figure 3. It can be seen that all lignins displayed a prominent absorption peak around 283 nm, which can be attributed to the π→π* electron transition of the aromatic ring, a characteristic of aromatic ring absorption bands. Additionally, weaker absorption peaks observed near 318 nm are associated with π→π* transitions in lignin units with C_α_ = C_β_ linkages, which are conjugated with the aromatic ring, as well as n→π* transitions in lignin units containing C_α_ = O groups that are conjugated with the aromatic ring [44]. These peaks may also correspond to conjugated phenolic units in ferulic acid, *p*-coumaric acids, or hydroxycinnamic acid-type structures present in lignins [45].

The sun emits electromagnetic radiation across three ultraviolet (UV) wavelength regions. The shortest wavelengths, UVC (100–280 nm), are absorbed by the atmosphere, while the mid-wavelengths (UVA, 280–320 nm) and longer wavelengths (UVB, 320–400 nm) reach the Earth’s surface. Overexposure to UVB can cause sunburn, while UVA penetrates deeper into the skin. Both UVA and UVB can cause DNA damage and increase the risk of cancer in humans. In addition, they significantly contribute to the aging process, leading to the discoloration, yellowing, and degradation of the mechanical properties of polymer materials [46,47]. Therefore, researching and applying UV shielding agents is essential to mitigate the harmful effects of UV radiation [48].

**Figure 4:**
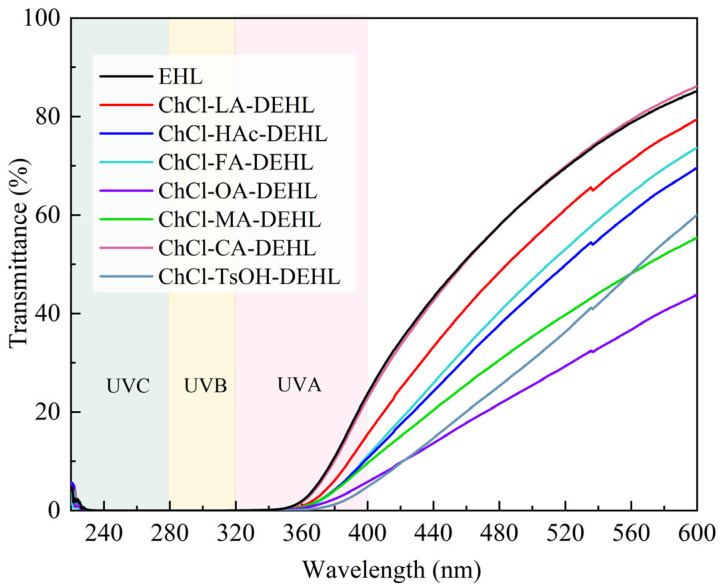
Ultraviolet–visible transmission spectra of EHLs and DEHLs.

The transmittance of EHLs and DEHLs under UV–visible light was analyzed, and the results are shown in Figure 4. The data showed that both EHLs and DEHLs possessed certain UV absorption and shielding effects, especially in the UVB stage, where the UV transmittance was almost 0. In the UVA stage, ChCl-TsOH-DEHL exhibited the highest UV shielding ability, with a UV transmittance of only 4.83% at 400 nm, followed by ChCl-OA-DEHL. The weakest UV shielding effects were observed for ChCl-CA-DEHL and EHL. In addition, it can be found that DEHL, which showed higher UVC shielding capacity, also exhibited greater shielding capacity in the visible range (400–600 nm).

### 3.5. Molecular Weight of Lignins

**Figure 5:**
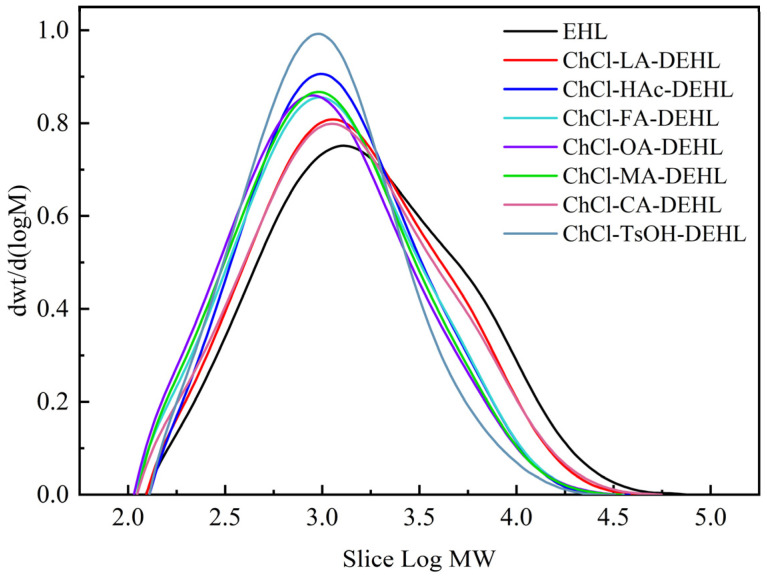
Molecular weights of EHLs, DEHLs, and BHT.

The molecular weight of lignins is closely associated with their physicochemical properties. In order to investigate the effects of different acidic DESs treatments on the molecular weight of EHLs, the molecular weights of EHLs and DEHLs were examined by gel permeation chromatography (GPC). The results are shown in Figure 5 and Table 3. As can be seen from the table, the weight-average molecular weight (*M*_w_), number-average molecular weight number (*M*_n_), and dispersion coefficient (*M*_w_/*M*_n_) of DEHLs were all lower than those of EHLs. This indicates that treatment of EHLs with acidic DESs under the experimental conditions mainly resulted in depolymerization, which disrupted the inter-unit bonding between the basic structural units (e.g., *β*-*O*-4 linkage) of the lignins, consequently decreasing the molecular weight of EHLs [49]. In addition, the dispersion coefficient of DEHLs was lower compared to that of EHLs, indicating a more uniform fragmentation of the lignins. Of the samples analyzed, ChCl-TsOH-DEHL had the lowest *M*_w_ (1586 g/mol) and *M*_W_/*M*_n_ (2.28), while ChCl-OA-DEHL had the smallest *M*_n_ (639 g/mol).

### 3.6. Total ArOH Content of Lignins

**Figure 6:**
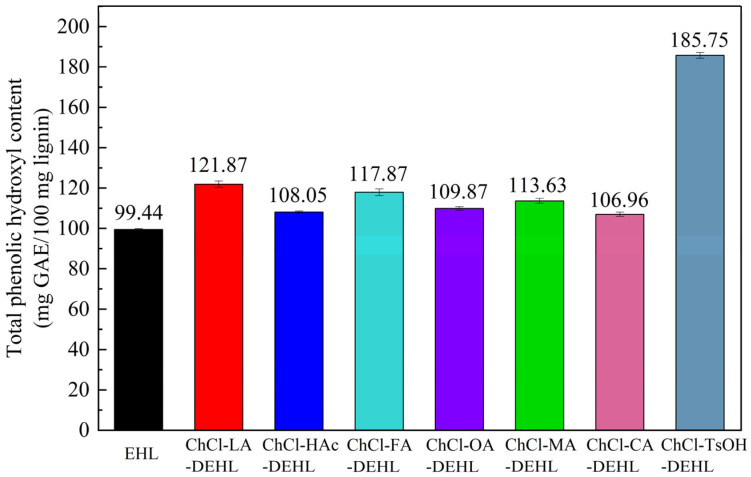
Total ArOH content of EHLs and DEHLs.

The total ArOH content of EHLs and DEHLs is shown in Figure 6. From the figure, it can be seen that the total ArOH content of DEHLs was higher than that of the untreated EHLs. Notably, ChCl-TsOH-DEHL had the highest total ArOH content, measuring 185.75 mg GAE/100 mg lignin. In contrast, ChCl-CA-DEHL had the lowest total ArOH content at 106.96 mg GAE/100 mg lignin. However, this value still surpassed the 99.44 mg GAE/100 mg lignin found in EHLs. The increase in ArOH content observed in DEHLs can be attributed to the disruption of the *β*-O-4 ether bond in EHLs by DESs, resulting in the formation of new ArOH groups [50].

### 3.7. Thermal Stability of Lignins

**Figure 7:**
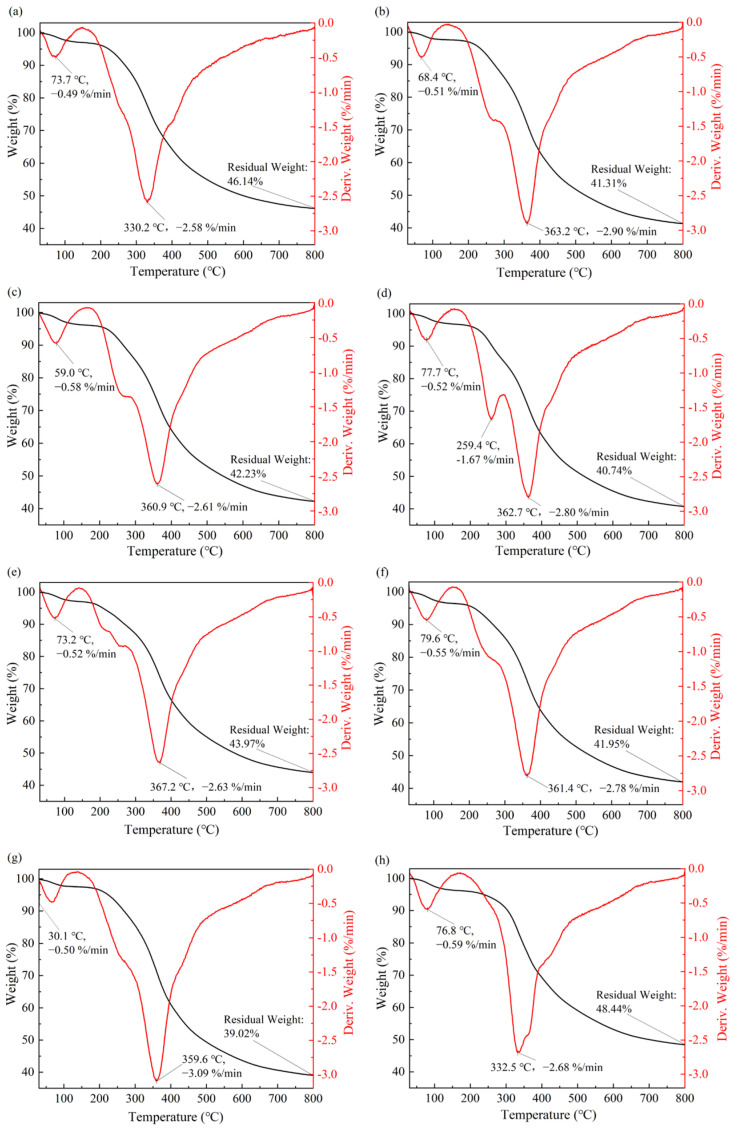
TG curves and DTG curves of EHLs and DEHLs: (**a**–**h**) are the TG curves and DTG curves of EHL, ChCl-LA-DEHL, ChCl-HAc-DEHL, ChCl-FA-DEHL, ChCl-OA-DEHL, ChCl-MA-DEHL, ChCl-CA-DEHL, and ChCl-TsOH-DEHL, respectively.

To analyze the effect of DESs treatment on the thermal stability of EHLs, the thermal stability of EHLs and DEHLs was tested using a thermogravimetric analyzer in the temperature range of 30–800 °C. The results of the thermogravimetric weight-loss (TG) curves of lignin and their first-order derivatives (DTG) curves are shown in Figure 7. The thermal decomposition of all lignin samples can be roughly divided into three stages.

The first stage occurred between 30 and 150 °C. This stage primarily involved the removal of adsorbed water in lignins and the degradation of low-molecular-weight lignins, resulting in a weight loss of about 3%.

The second stage occurred between 150 and 600 °C. This phase represented the rapid pyrolysis of the lignin samples. During this stage, mass loss was primarily due to the degradation of lignin phenylpropane polymers, particularly the breakdown of the *β*-*O*-4 basic structural unit and the cleavage of lignin side chains [51,52]. At this stage, the peak degradation rate for EHL was observed at an inflection temperature of 330.2 °C, corresponding to a degradation rate of 2.58%/min. It is widely recognized that ball-milled lignins closely resemble the structure of natural lignins found in plants, characterized by a relatively rich presence of easily degradable *β*-*O*-4 ether bonds. Study [53] demonstrated that the temperature range for ball-milled lignins to achieve their maximum degradation rate is approximately between 316 and 330 °C. Notably, the inflection temperature for EHL aligned closely with that of ball-milled lignins, suggesting that the milder extraction conditions for EHLs better preserve the original lignin structure [54]. The inflection point temperatures of ChCl-LA-DEHL, ChCl-HAc-DEHL, ChCl-FA-DEHL, ChCl-OA-DEHL, ChCl-MA-DEHL, ChCl-CA-DEHL, and ChCl-TsOH-DEHL increased from 330.2 °C for EHLs to 363.2 °C, 360.9 °C, 362.7 °C, 367.2 °C, 361.4 °C, 359.6 °C, and 332.5 °C, respectively, indicating that the thermal stability of EHLs improved after treatment with DESs. Specifically, the thermal stability of the six DESs-treated DEHLs increased by about 30 °C, except for ChCl-TsOH-DEHL, which showed a slight increase of 2.3 °C. The increase in the thermal stability of DEHLs during the second stage could be attributed to the cleavage of the less thermally stable *β*-*O*-4 ether bonds during the DES treatment. Furthermore, treatments with ChCl-LA, ChCl-HAc, ChCl-FA, ChCl-OA, ChCl-MA, and ChCl-CA may induce esterification reactions of certain hydroxyl groups in lignin, which further contributes to the improvement of the thermal stability of DEHLs [55]. Additionally, with the exception of ChCl-TsOH, other DESs treatments resulted in enhanced shoulder peaks at lower temperatures in DEHLs, especially ChCl-FA-DEHL, which exhibited a pronounced shoulder peak at 259.4 °C, with a corresponding degradation rate of 1.67%/min. This suggests the presence of more thermally unstable regions in DEHLs following treatment with these six acidic DESs [56].

The third stage of lignin thermal degradation occurred between 600 and 800 °C. This stage is characterized by the carbonization of the relatively stable aromatic ring structure. Due to the stability of these aromatic ring structures, lignins lost weight slowly during this stage. As the temperature continued to rise, a series of reactions, including carbon–carbon bond breakage, occurred within the aromatic rings of lignin, eventually leading to its degradation into small molecules such as CO_2_, CO, and H_2_O [57]. At 800 °C, the char yield of EHL was 46.14%. However, with the exception of ChCl-TsOH-DEHL, all other DESs treatments resulted in a reduction in the char yield of EHL at 800 °C. Meanwhile, the char yield of ChCl-TsOH-DEHL increased from 46.14% fo EHL to 48.44% at 800 °C.

It can be found that different DESs treatments have varying effects on the thermal stability of EHLs. All seven DESs treatments significantly improved the thermal stability of EHLs within 150 °C to 600 °C. With the exception of ChCl-TsOH-DEHL, the other DESs treatments reduced the char yield of lignins at 800 °C to some extent. Overall, ChCl-TsOH treatment enhanced the thermal stability of EHL, helping to preserve the integrity of its chemical structure and functional properties at elevated temperatures.

### 3.8. Antioxidant Capacity of Lignins

DPPH (2,2-diphenyl-1-picrylhydrazyl) is a stable radical commonly used to assess the free radical scavenging ability of various substances. When dissolved in methanol, DPPH^•^ shows a characteristic dark purple color and displays strong absorption at 517 nm. Upon reaction with antioxidants, the absorption at 517 nm decreased. The antioxidant activity of a substance can be assessed by calculating the percentage inhibition of DPPH^•^, which is determined through absorbance measurements at 517 nm using a UV spectrophotometer.

**Figure 8:**
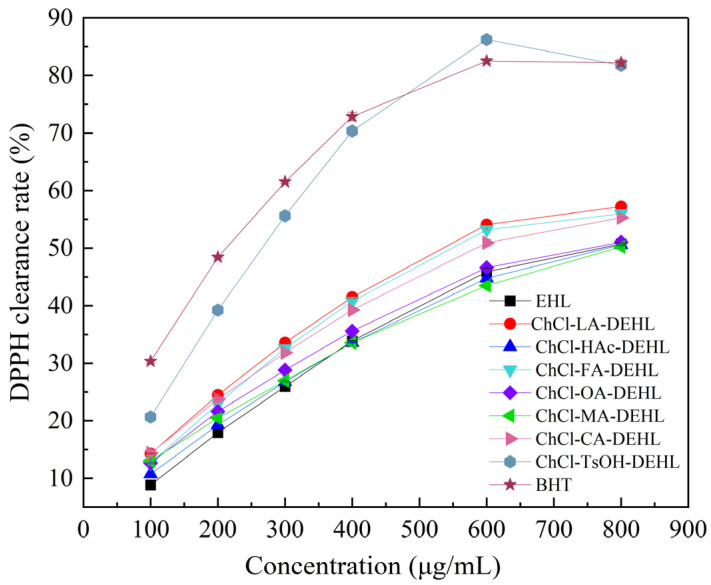
Scavenging capacity of EHLs and DEHLs for DPPH^•^.

The scavenging effect of EHLs and DEHLs on DPPH^•^ is shown in Figure 8. Using the commercial synthetic food antioxidant butylated hydroxytoluene (BHT) as a control, the antioxidant capacity of different lignins was analyzed. The results showed that, at lower concentrations, the DPPH^•^ scavenging ability of lignins generally increased with concentration. However, the antioxidant capacity of lignin did not show a consistent positive correlation with its concentration changes. This might be due to the tendency of lignin to aggregate at higher concentrations, thereby reducing the number of active groups available to react with DPPH^•^ [58]. Under the conditions studied, with the exception of ChCl-MA and ChCl-HAc, the other five DESs treatments enhanced the antioxidant activity of lignin to a certain extent. Overall, the antioxidant activities of EHLs and DEHLs were lower than that of the commercial antioxidant BHT. Interestingly, the antioxidant activity of ChCl-TsOH-DEHL at higher concentrations surpassed that of BHT, achieving a DPPH^•^ scavenging rate of 86.22% at a concentration of 600 μg/mL, compared to BHT’s rate of 82.50% at the same concentration.

Therefore, DEHLs with enhanced antioxidant properties show promise as natural antioxidants for various applications, including skin care products, food packaging, composites, and pharmaceutical materials.

**Figure 9:**
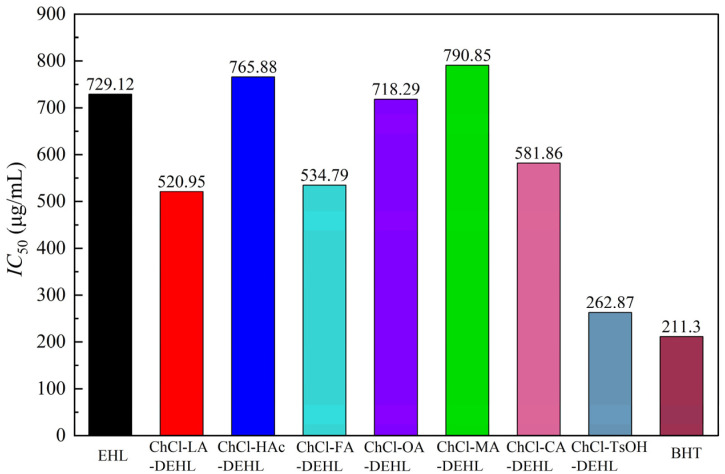
*IC*_50_ value of EHLs, DEHLs, and BHT.

The half-maximal inhibitory concentration (*IC*_50_) is defined as the concentration of a sample required for the free radical clearance rate to reach 50%, serving as a crucial indicator of the antioxidant activity of the sample [59]. The *IC*_50_ values for EHLs, DEHLs, and BHT in relation to DPPH^•^ are presented in Figure 9. A lower *IC*_50_ value indicates the stronger ability of the material to scavenge DPPH^•^. The results reveal that the highest *IC*_50_ value was observed for ChCl-MA-DEHL (783.55 μg/mL), followed by ChCl-HAc-DEHL (765.88 μg/mL) and EHLs (729.12 μg/mL). The lowest *IC*_50_ value was found for ChCl-TsOH-DEHL (262.87 μg/mL), which was closest to the *IC*_50_ value of BHT (211.30 μg/mL). This finding suggests that the antioxidant capacity of EHLs was significantly improved after treatment with ChCl-TsOH. Based on the previous analysis of the chemical structure of DEHL, this increase in antioxidant activity may be due to the reduction in molecular weight and the increase in ArOH content after treatment with ChCl-TsOH compared to EHLs [60].

**Figure 10:**
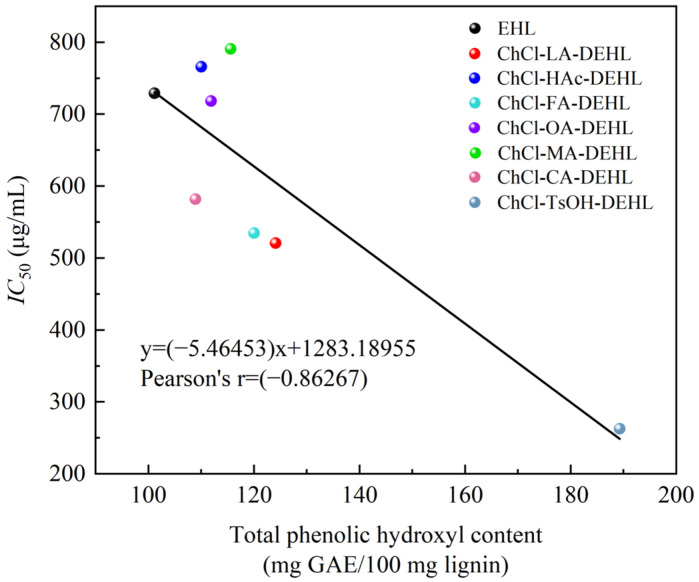
Relationship between total ArOH content of lignins and their half-inhibitory concentration of DPPH free radicals.

In order to analyze the relationship between the ArOH content of EHLs and DEHLs and their antioxidant activity, relationship curves were plotted using the total ArOH content of EHLs and DEHLs as the horizontal axis and their *IC*_50 DPPH_ value against DPPH^•^ as the vertical axis. The results are shown in Figure 10. It can be seen that there is a linear relationship between the total ArOH content of lignin and the *IC*_50 DPPH_ value, with a Pearson correlation coefficient of −0.86267. This suggests a strong negative linear correlation between the total ArOH content of lignins and their *IC*_50 DPPH_ values. Nsimba et al. [61] reported linear correlation coefficients (R^2^) of 0.7983 and 0.8185 for the ArOH and methoxyl contents of lignins, respectively, in relation to their *IC*_50 DPPH_ values. These findings suggest that there is a strong linear correlation between the ArOH and methoxyl contents of lignin and their free radical scavenging ability.

Overall, ChCl-TsOH-DEHL exhibited the highest ArOH content, antioxidant activity and thermal stability among the several lignins studied in this research. Infrared analysis, molecular weight assessments and thermogravimetric analysis indicated that the treatments of EHLs with ChCl-LA, ChCl-HAc, ChCl-FA, ChCl-OA, ChCl-MA, and ChCl-CA resulted in the cleavage of some ether bonds between the basic structural units of lignins. This degradation led to a decrease in the molecular weight of DEHLs, an increase in ArOH content, and an improvement in thermal stability within the range of 150–600 °C. However, the carboxylic acids present in these DESs undergo esterification reactions with some of the hydroxyl groups in the regenerated lignins, which consequently reduced the ArOH content. As a result, the enhancement of antioxidant activity of the regenerated lignins following these DES treatments was not as pronounced as that of the ChCl-TsOH treatment.

## 4. Conclusions

In this study, seven acidic DESs were prepared using ChCl as the hydrogen bond acceptor and lactic acid, acetic acid, formic acid, oxalic acid, malic acid, citric acid, and *p*-toluenesulfonic acid as hydrogen bond donors. These seven acidic DESs were utilized to treat EHL.

The results showed that the yield of DEHLs treated with several acidic DESs ranged from 92.03% to 94.14%. DEHLs essentially retained the aromatic structures of EHLs, and the analysis suggested that lignins may have undergone a sulfonation reaction during treatment with ChCl-TsOH. With the exception of ChCl-CA, treatment with the other acidic DESs enhanced the UV shielding ability of the lignins, with ChCl-TsOH-DEHL exhibiting the best UV shielding and antioxidant performance. The acidic DESs treatments significantly reduced both the *M*_w_ and *M*_n_ of EHLs. Of the treatments, ChCl-TsOH-DEHL showed the lowest molecular weight, with *M*_w_ and *M*_n_ measured at 3223 g/mol and 939 g/mol, respectively. Moreover, treatment with acidic DESs increased the phenolic content of EHL to varying degrees. Notably, ChCl-TsOH treatment resulted in the highest increase in the ArOH content of DEHLs, raising it from 99.44 mg GAE/100 mg lignin to 185.75 mg GAE/100 mg lignin. Except for ChCl-MA and ChCl-HAc, the antioxidant activity of DEHLs improved after treatment with the other acidic DESs. The *IC*_50 DPPH_ values of ChCl-LA-DEHL, ChCl-FA-DEHL, ChCl-OA-DEHL, ChCl-CA-DEHL, and ChCl-TsOH-DEHL decreased from the 729.12 μg/mL of EHLs to 520.95, 534.79, 718.29, 581.86, and 262.87 μg/mL, respectively, indicating that all these DESs treatments were effective in improving the antioxidant activity of EHLs. In particular, the antioxidant activity of ChCl-TsOH-DEHL at a concentration of 600 μg/mL surpassed that of the commercial antioxidant BHT. Furthermore, the DPPH^•^ scavenging capacity of both EHLs and DEHLs exhibited a strong negative correlation with their ArOH content. In conclusion, this study found that the acidity of the DESs had little effect on the yield, chemical structure, molecular weight, and antioxidant activity of DEHLs, while the type of acid in DESs had a significant impact on the properties of DEHLs. In particular, treatment with ChCl-TsOH significantly improved the antioxidant activity of DEHLs, thereby broadening the potential applications of EHLs.

## Figures and Tables

**Table 1:** Yield of DEHL treated with different acidic DESs.

DESs Type	pH Value of the DESs	DEHL Yield/%
ChCl-LA	3.37 (0.06)	92.87
ChCl-HAc	1.10 (0.02)	92.03
ChCl-FA	−0.04 (0.04)	92.16
ChCl-OA	<−2.00	93.00
ChCl-MA	−0.62 (0.05)	94.14
ChCl-CA	−1.43 (0.11)	92.82
ChCl-TsOH	−1.92 (0.08)	92.44

**Table 2:** The O/C atomic ratio of EHLs and DEHLs.

Lignin	O1s (%)	C1s (%)	O/C
EHL	19.27	80.73	0.239
ChCl-LA-DEHL	22.04	77.96	0.283
ChCl-HAc-DEHL	20.21	79.79	0.253
ChCl-FA-DEHL	20.09	79.91	0.251
ChCl-OA-DEHL	20.75	79.25	0.262
ChCl-MA-DEHL	21.38	78.62	0.272
ChCl-CA-DEHL	20.86	79.14	0.264
ChCl-TsOH-DEHL	19.98	80.02	0.250

**Table 3:** Molecular weight and polydispersity of EHLs and DEHLs.

Lignin	*M*_W_ (g/mol)	*M*_n_ (g/mol)	*M*_W_/*M*_n_
EHL	3223	939	3.43
ChCl-LA-DEHL	2528	839	3.01
ChCl-HAc-DEHL	1901	761	2.50
ChCl-FA-DEHL	1912	691	2.80
ChCl-OA-DEHL	1780	639	2.78
ChCl-MA-DEHL	1824	671	2.72
ChCl-CA-DEHL	2538	786	3.23
ChCl-TsOH-DEHL	1586	697	2.28

## Data Availability

Data are contained within the article.
